# Factors Associated with Physician Agreement on Verbal Autopsy of over 27000 Childhood Deaths in India

**DOI:** 10.1371/journal.pone.0009583

**Published:** 2010-03-08

**Authors:** Shaun K. Morris, Diego G. Bassani, Rajesh Kumar, Shally Awasthi, Vinod K. Paul, Prabhat Jha

**Affiliations:** 1 Centre for Global Health Research, Li Ka Shing Knowledge Institute, St. Michael's Hospital, Toronto, Canada; 2 Division of Infectious Diseases, Department of Pediatrics, Hospital for Sick Children, University of Toronto, Toronto, Canada; 3 Dalla Lana School of Public Health, University of Toronto, Toronto, Canada; 4 Child Health and Evaluative Sciences, Hospital for Sick Children, Toronto, Canada; 5 School of Public Health, Post Graduate Institute of Medical Education, Chandigarh, India; 6 Department of Pediatrics, King George's Medical University, Lucknow, India; 7 Department of Pediatrics, All India Institute of Medical Sciences, New Delhi, India; University of Oxford, United Kingdom

## Abstract

**Introduction:**

Each year, more than 10 million children younger than five years of age die. The large majority of these deaths occur in the developing world. The verbal autopsy (VA) is a tool designed to ascertain cause of death in such settings. While VA has been validated against hospital diagnosed cause of death, there has been no research conducted to better understand the factors that may influence individual physicians in determining cause of death from VA.

**Methodology/Principal Findings:**

This study uses data from over 27,000 neonatal and childhood deaths from The Million Death Study in which 6.3 million people in India were monitored for vital status between 1998 and 2003. The main outcome variable was physician agreement or disagreement of category of death and the variables were assessed for association using the kappa statistic, univariate and multivariate logistic regression using a conceptual hierarchical model, and a sensitivity and specificity analysis using the final VA category of mortality as the gold standard. The main variables found to be significantly associated with increased physician agreement included older ages and male gender of the deceased. When taking into account confounding factors in the multivariate analysis, we did not find consistent significant differences in physician agreement based on the death being in a rural or urban area, at home or in a health care facility, registered or not, or the respondent's gender, religion, relationship to the deceased, or whether or not the respondent lived with the deceased.

**Conclusions/Significance:**

Factors influencing physician agreement/disagreement to the greatest degree are the gender and age of the deceased; specifically, physicians tend to be less likely to agree on a common category of death in female children and in younger ages, particularly neonates. Additional training of physician reviewers and continued adaptation of the VA itself, with a focus on gender and age of the deceased, may be useful in increasing rates of physician agreement in these groups.

## Introduction

Each year, more than 10 million children younger than five years of age die [1], [2]. The ability to accurately measure causes of these deaths is the foundation of global childhood health interventions, policy, and research[3], [4]. However, the majority of childhood deaths occur in the developing world and outside of the formal health care sector. These deaths are unlikely to be registered, thus introducing significant limitations any inferences drawn from vital registration systems in these regions. The verbal autopsy (VA) is a tool designed to ascertain cause of death in such settings. The VA relies on the assumption that various causes of death have symptoms and signs that can be recalled and accurately reported by care givers and family members during a standardized interview by a trained, generally non-medical, fieldworker. It also relies on the assumption that the symptoms and signs of different clinical conditions are sufficiently distinct so as to permit one cause of death to be distinguished from others.

Though efforts have been made at standardizing VA instruments [5], published research using VA utilize differing methodologies. While some VA studies utilize data or expert derived algorithms[6], symptom pattern methodology[7], or probabilistic methods[8], [9] many utilize two or more trained physicians to review VA forms and determine a cause of death which is then coded using the World Health Organization (WHO) International Classification of Disease 10 (ICD-10) [10].

To date, validation studies of VA using physician coders have generally compared the physician identified codes to known hospital diagnoses or death certificates as the gold standard[11]–[14] [15], [16]. However, this group of patients may not be representative of the entire population, especially in settings where access to health care is limited. Little is known about the factors that contribute to physician agreement in VA studies using multiple physician coders. Understanding these factors is of critical importance to developing a better understanding of how accurately the many and diverse studies based on the verbal autopsy may reflect actual underlying patterns of mortality. Thus, the objective of this study is to understand if factors specific to either the deceased or to the respondent of the VA are associated with physician reviewer's agreement on cause of death.

## Methods

This study uses data from the Million Death Study (MDS) [17]. In brief, 6.3 million people in 1.1 million nationally representative Indian households were monitored for vital events between 1998 and 2003. An average of 150 households were selected from each of 6671 sampling units which comprise all 35 states and union territories of India. The sample units were randomly selected to be reflective of population at the state level. For each household death that occurred, a standard VA questionnaire, called RHIME (Routine, Reliable, Representative and Re-sampled Household Investigation of Mortality with Medical Evaluation), that uses both an open-ended narrative and close-ended structured questions was administered[18]. Interviewers were Registrar General of India surveyors, with knowledge of local language(s), and trained in the RHIME instrument. Using an Internet-based application, two independent physicians reviewed each completed RHIME form and assigned a single cause of death using the ICD-10 [10] as well as a list of key clinical words to reflect how they arrived at their diagnosis. If the physicians initially agreed on the cause of death, this cause of death was finalized. If the two physicians initially disagreed on the cause of death, their respective keywords used to determine the cause of death are exchanged and an attempt is made to reconcile to a common ICD-10 code. If the two physicians were able to reconcile to a common ICD-10 code, this cause of death became finalized at this stage. If the two physicians continue to disagree following the reconciliation stage, a third, senior physician adjudicates and determines the final cause of death. In order to ensure reproducibility, a random 5% sample of completed RHIME's are chosen and repeated in their entirety, from data collection to physician coding. Further details about the MDS methodology are described elsewhere [17] For this study, we used MDS data from 2001 to 2003 and our analysis grouped ICD-10 codes into broad categories (7 categories for neonates 0 to 28 days and 9 for infants/children 29 days to 14 years) which made up approximately 80% of all deaths in each age group. The categories used for the neonatal group are: low birth weight/pre-term birth, birth asphyxia/birth trauma, vaccine preventable diseases, diarrheal diseases, congenital anomalies, other infectious diseases, and other perinatal conditions. The categories used for the infant/childhood group are: acute respiratory infections, diarrheal diseases, malaria, vaccine preventable diseases, central nervous system infections, other infectious diseases, injuries, nutritional diseases, and other non-infectious diseases. The ICD-10 codes contained in each of these categories can be seen in Tables 1 and 2.

**Table 1:** ICD-10 codes in neonatal categories of death.

Neonatal Category	ICD-10 Codes
Low Birth Weight/Preterm	P05, P07, P52, P77
Other Infections	All other A and B codes not listed in the table, G00-G09, H60, H65-H68,H70-H71, I30, I32-I33, I39-I41, J00-J06, J09-J18, J20-J22, J32, J36,J85-J86, K65, K81, L00-L04, M00-M01, M60, M86, N10, N30, N34,N41, N49, N61, P23, P36-P39, R50, U04
Birth Asphyxia/Trauma	P02-P03, P10-P15, P20-P22, P24-P29, P50, P90, P91
Vaccine Preventable	A33-A37, A80, B01, B03, B05-B06, B26, B91
Diarrhea	A00-A09
Congenital Anomalies	Q00-Q99
Other Conditions	All other codes

**Table 2:** ICD-10 codes in childhood categories of death.

Childhood Category	ICD-10 Codes
Acute Respiratory Infections	H65-H68, H70-H71, J00-J06, J09-J18, J20-J22, J32, J36, J85-J86, P23, U04
Diarrhea	A00-A09
Malaria	B50-B54
Vaccine Preventable	A33-A37, A80, B01, B03, B05-B06, B26, B91
CNS Infections	A81-A89, G00-G09
Other Infectious Diseases	All other A and B codes not listed in the table, H60, H65-H68, I30, I32-I33,I39-I41, K65, K81, L00-L04, L08, M00-M01, M60, M86, N10, N30, N34, N41,N61, P36-P39, R50
Injuries	All S, T, V, W, X, and Y codes
Nutritional	D50-D53, E00-E02, E40-E46, E50-E56, E58-E61, E63-E64, X53-X54
Other Non-Infectious Diseases	All other codes

We compared the defined categories of causes of death coded by each physician and percent agreement using the kappa statistic[19]. The 95 percent confidence interval for the kappa statistic was calculated using the bootstrap method for polychotomous variables with bias correction [20]. Analyses were conducted on the entire infant/childhood data set as well as stratified by age category (29 days to 364 days, 1 year to 4 years, and 5 years to 14 years).

Sensitivity and specificity of initial physician cause of death diagnosis was calculated using standard methods with the final RHIME cause of death as the gold standard. This portion of the analysis is to be interpreted only with the understanding that the final RHIME cause of death is not independent from the initial physician diagnoses.

In order to determine how well the household members themselves were able to identify the cause of death, we calculated the sensitivity and specificity of cause of death as identified by the household versus the final cause of death as assigned by the RHIME. The household's determination of cause of death was collected on Form 12 which served as an independent source of crude cause of death estimates. These deaths were reported by the household to the SRS surveyor for the period of time from 1998 to 2003. The SRS surveyor classified the household identified cause of death into one of 79 specific causes. We then collapsed the 79 causes into the same above described categories of death for both neonates and older children and then deaths registered in Form 12 were matched to the same individuals on the RHIME for comparison.

The crude association between demographic, socioeconomic, geographic, and other factors potentially associated with physician agreement was tested using a univariate logistic regression model. To adequately adjust for confounders, multivariate logistic regression was also used to analyze the association between these factors and the outcome of physician agreement following the intial stage of review of the verbal autopsy. The modeling procedure used an a priori conceptual hierarchical model organized into three blocks of variables[21]. The first, distal block included geographic factors (Empowered Action Group (EAG) region plus Assam, geographic region, and urban or rural place of residence), the second, middle block contained sociodemographic factors of the respondent (whether the respondent lived with the deceased and the respondent's gender, education, religion, and relationship to the deceased), and the third, proximal block, contained factors specific to the deceased individual (gender of the deceased, the location of the death, and whether or not the death was registered). The model was adjusted using backwards elimination. In order for a variable to be considered as a potential confounder and to be retained in the multivariate model, it had to show a p-value <0.20 in the likelihood ratio test. Finally, variables were considered to be significantly associated with the outcome for p-value <0.05 in the likelihood ratio test.

All statistical analysis was performed using Stata SE 10 [22].

Ethics approval for the MDS was obtained from the Indian Council of Medical Research and the Postgraduate Institute of Medical Education and Research, Chandigarh, India and St. Michael's Hospital, Toronto, Ontario, Canada.

## Results

**Figure 1:**
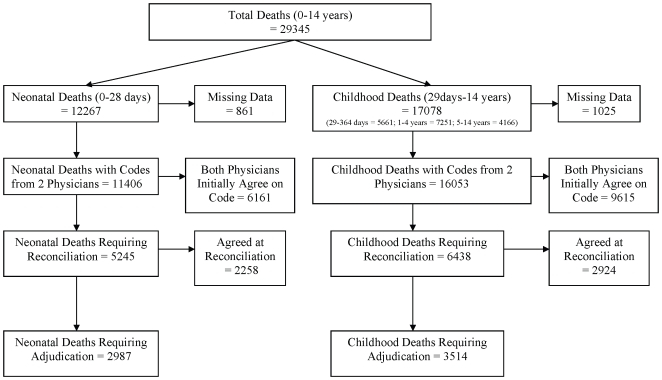
Flow Diagram of Million Death Study Childhood (<15 years) Deaths.

There were a total of 29345 deaths in children under the age of 15 during the study period (Figure 1). Of these, the RHIME forms for 27459 (11406 neonatal deaths and 16053 childhood deaths) were coded by two physicians and were thus included in the analysis of physician agreement.

The kappa statistic is used to analyze the agreement between the two physicians taking into account the likelihood they will agree on common category of death based on chance alone. The kappa analysis is shown in Table 3. The strength of agreement of the kappa coefficient is shown in Table S1. For neonates, the overall physician agreement was 64.9% with a kappa of 0.56 (95% CI 0.55–0.57). When the neonatal age group was broken down into the first week of life and weeks 2 to 4, physician agreement increased with older neonates (days of life 0 to 7 physician agreement 64.0% and kappa 0.54 (95% CI 0.54–0.55); days of life 8 to 28 physician agreement 68.3% and kappa 0.58 (95% CI 0.56–0.59)). There was little significant variation in the kappa statistic across the various strata including the gender of the deceased, whether the place of residence was in an urban or rural area, and whether the RHIME respondent lived with the deceased or not. Physicians were more likely to agree when the RHIME respondent was a non-parental relative to the deceased (generally a sibling, grandparent, aunt, or uncle) than a parent. Physicians were slightly less likely to agree on a cause of death when the place of death was a health facility compared to the home.

**Table 3:** Kappa analysis by age category.

			Neonates 0-28 days			Infants 29 days to <1 year			Children 1 to 4 years			Children 5 to 14 years	
Variable	Sub-Categories	Number	% Agree	Kappa	Number	% Agree	Kappa	Number	% Agree	Kappa	Number	% Agree	Kappa
Overall		10688	64.9	0.56	5251	71.8	0.64	6910	72.1	0.67	3873	75.9	0.71
Gender	Male	5898	64.3	0.55	2499	72.6	0.65	3074	73.1	0.68	1914	78.6	0.74
	Female	4703	65.6	0.57	2748	71.1	0.64	3835	71.2	0.66	1959	73.2	0.68
Rural/Urban	Rural	9728	65.0	0.56	4692	71.9	0.64	6355	72.1	0.67	3485	75.9	0.71
	Urban	958	63.9	0.54	557	71.3	0.64	554	71.7	0.66	388	75.3	0.69
Live With Status	Yes	8403	64.7	0.56	4162	72.4	0.65	5491	71.9	0.66	3030	75.2	0.70
	No	1632	65.6	0.56	666	72.4	0.65	881	73.7	0.69	541	79.3	0.74
Respondent Relation	Parent	5981	63.9	0.55	2983	72.6	0.65	4014	71.8	0.66	2393	76.0	0.71
	Other Relative	4160	66.2	0.58	1942	70.4	0.63	2424	72.4	0.67	1209	75.9	0.71
	Unrelated	370	67.0	0.59	192	73.4	0.67	327	73.1	0.68	186	74.7	0.69
Respondent Education	Less than Primary	6775	64.9	0.56	3419	71.2	0.64	4867	72.4	0.67	2544	75.7	0.71
	Primary	1183	64.5	0.55	606	72.1	0.65	661	69.1	0.64	414	75.1	0.70
	>Primary	2503	64.6	0.56	1125	73.6	0.67	1250	72.7	0.67	839	76.9	0.72
Respondent Gender	Male	4922	64.0	0.55	2470	71.9	0.65	3578	71.4	0.66	2063	76.6	0.72
	Female	5729	65.7	0.57	2675	72.0	0.65	3208	72.8	0.68	1725	75.0	0.70
Death Place	Home/Other	7875	65.1	0.56	4342	71.4	0.64	5825	72.0	0.67	3088	76.0	0.71
	Health Facility	2358	63.9	0.53	671	74.2	0.67	771	72.1	0.67	620	76.7	0.70
Religion	Hindu	8595	64.9	0.56	4172	71.9	0.64	5397	72.2	0.67	3003	76.0	0.71
	Muslim	1494	65.5	0.57	751	72.4	0.65	1067	72.7	0.68	535	74.0	0.69
	Other	599	63.6	0.54	328	69.8	0.61	446	69.3	0.63	335	77.3	0.73
Registration of Death	Yes	1556	66.5	0.57	794	73.4	0.66	1020	70.0	0.64	789	77.8	0.72
	No	5069	64.7	0.56	2439	70.7	0.63	3300	73.9	0.69	1672	74.4	0.70
EAG+Assam vs Non-EAG	EAG + Assam	6752	64.7	0.56	3317	72.3	0.65	4809	72.5	0.67	2479	75.1	0.71
	Non-EAG	3934	65.3	0.55	1932	71.1	0.63	2100	71.1	0.65	1394	77.3	0.72

Footnote: Unknown values omitted.

Physician agreement and the kappa statistic increase with child's age for the posteneonates. Overall agreement was 71.8% and kappa 0.64 (95% CI 0.63–0.66) in infants aged 29 days to under 1 year, agreement was 72.1% and kappa 0.67 (95% CI 0.66–0.68) in children aged 1 year to 4 years, and agreement was 75.9% and kappa 0.71 (95% CI 0.71–0.72) in older children aged 5 years to 14 years. In all three age categories, physicians were more likely to agree on causes of boy deaths than in girl deaths, although this difference was not statistically significant in the 29 days to less than 1 year group. For children aged 1 to 4 years, physicians were slightly more likely to agree in cases in which the death was not registered.

The sensitivity and specificity of an individual physician's determined cause of death was estimated following the initial review stage, compared to the final cause of death as determined by the RHIME instrument. These results may be seen in Table 4. There were 23098 neonatal and 34214 childhood physician determined categories of death (two physician codes per death). Due to the large number of observations, we utilized a 99 percent confidence interval around the sensitivity and sensitivity point estimates. We found that for neonates, the sensitivity of the initial physician coded cause of death was greater than 80% for all categories except for asphyxia/birth trauma (sensitivity 74.9%, 99% CI 73.2–76.5) and congenital malformations (79.0%, 99% CI 74.8–82.8). The specificity at this stage was greater than 93% for all categories. In children, the sensitivity of physician coding at the initial stage was less than 80% for the categories of central nervous system infections, malaria, nutritional diseases, and other infectious diseases. Consequently, the specificity for each of these conditions in each of the childhood age categories exceeded 98%. The specificity for all childhood categories at the initial stage of physician review was greater than 93%.

**Table 4:** Sensitivity and specificity of initial physician coded cause of death versus final RHIME cause by age category.

Age Category	Cause of Death Category	Sensitivity	99% CI	Specificity	99% CI
0 to 28 days	Low Birth Weight/Pre-Term	83.7	(82.4–84.9)	96.4	(96.0–96.8)
(n = 23098)	Other Infectious Diseases	83.2	(81.8–84.5)	95.7	(95.3–96.1)
	Asphyxia and Birth Trauma	74.9	(73.2–76.5)	95.3	(94.9–95.7)
	Other Perinatal Conditions	82.0	(80.1–83.8)	93.2	(92.7–93.6)
	Vaccine Preventable Diseases	83.7	(80.6–86.4)	99.3	(99.1–99.4)
	Diarrheal Disease	83.6	(79.6–87.1)	99.5	(99.3–99.6)
	Congenital	79.0	(74.8–82.8)	99.5	(99.4–99.6)
29 days to 1 yr	Acute Respiratory Infections	87.7	(86.2–89.0)	96.8	(96.3–97.3)
(n = 11322)	Non-infectious Conditions	86.2	(84.2–88.0)	93.9	(93.2–94.5)
	Diarrheal Diseases	89.7	(87.8–91.3)	98.0	(97.6–98.4)
	Injuries	88.4	(82.7–92.7)	99.7	(99.6–99.8)
	Other Infectious Diseases	65.3	(60.6–69.8)	98.1	(97.7–98.4)
	Malaria	79.4	(72.9–85.0)	99.2	(99.0–99.4)
	Vaccine Preventable Dis	81.8	(76.5–86.4)	99.3	(99.1–99.5)
	Nutritional Diseases	76.0	(71.1–80.4)	98.8	(98.0.5–99)
	CNS Infections	65.4	(58.2–72.2)	99.2	(99.0–99.4)
1 to 4 yr	Acute Respiratory Infections	87.0	(85.4–88.5)	97.5	(97.0–97.8)
(n = 14502)	Non-infectious Conditions	84.8	(82.7–86.7)	94.1	(93.5–94.6)
	Diarrheal Diseases	90.7	(89.3–91.9)	97.5	(97.1–97.9)
	Injuries	95.4	(93.6–96.8)	99.7	(99.6–99.8)
	Other Infectious Diseases	65.1	(61.0–69.0)	98.2	(97.9–98.5)
	Malaria	79.0	(75.3–82.5)	98.8	(98.6–99.1)
	Vaccine Preventable Dis	86.0	(83.0–88.7)	99.2	(98.9–99.4)
	Nutritional Diseases	69.8	(64.9–74.4)	99.1	(98.8–99.3)
	CNS Infections	68.9	(63.7–73.8)	99.1	(98.8–99.3)
5 to 14 yr	Acute Respiratory Infections	82.1	(78.4–85.4)	98.4	(98.0–98.8)
(n = 8390)	Non-infectious Conditions	87.8	(85.6–89.7)	94.4	(93.6–95.1)
	Diarrheal Diseases	90.1	(87.9–92.1)	98.2	(97.7–98.6)
	Injuries	97.4	(96.2–98.3)	99.5	(99.2–99.7)
	Other Infectious Diseases	70.4	(65.4–75.0)	98.4	(98.0–98.8)
	Malaria	83.5	(79.6–86.9)	98.8	(98.4–99.1)
	Vaccine Preventable Dis	84.3	(78.7–88.8)	99.5	(99.3–99.7)
	Nutritional Diseases	70.9	(58.6–81.4)	99.6	(99.4–99.8)
	CNS Infections	72.7	(66.9–78.0)	99.0	(98.7–99.3)

A univariate analysis was performed to analyze crude associations and a hierarchical multivariate logistic regression (Table 5) was performed to adequately adjust for confounders. In both analyses, a p-value<0.05 was deemed to be significant. For neonates in the univariate analysis, using parental respondent as baseline, a higher degree of physician agreement at the initial stage was associated with non-grandparent, non-sibling relative respondents (OR 1.17, 95% CI 1.06–1.30), female respondents (OR 1.10, 95% CI 1.02–1.17), non-Hindu and non-Muslim religion (OR 1.20, 95% CI 1.01–1.42), and registered deaths (OR 1.14, 95% CI 1.01–1.28). After adjusting for confounders in the multivariate analysis, respondent gender being female (OR 1.09, 95% CI 1.00–1.18) and, and the respondent being a grandparent (OR 1.14, 95% CI 1.02–1.27) or other non-parent, non-sibling relative (OR 1.11, 95% CI 1.00–1.18) were significantly associated with increased physician agreement. In infants aged 29 days to less than 1 year, the death being registered was significantly associated with increased physician agreement (OR 1.22, 95% CI 1.02–1.45). However, in the multivariate analysis in this age group, only respondent education greater than primary level was associated with physician agreement. The univariate analysis in children aged 1 year to 4 years found that death place being outside of a health facility and the home (generally as a result of a trauma or injury) was associated with increased physician agreement (OR 1.55, 95% CI 1.26–1.90). Death place outside the home or health care centre continued to be associated with increased physician agreement in this age group in the multivariate model. In the univariate analysis for older children, aged 5 to 14 years, there was greater agreement for the deaths of boys (OR 1.26, 95% CI 1.14–1.36), death outside of the home or health care facility (OR 2.62, 95% CI 2.02–3.41), registered deaths (OR 1.25, 95% CI 1.03–1.53), and in EAG states plus Assam (OR 1.18, 95% CI 1.01–1.37). In the multivariate analysis, physician agreement was also found to be greater for the deaths of boys and when the death occurred outside the home or health facility. Because we believe deaths due to injuries to be more likely to occur outside of the home or health facility and also more likely to be agreed upon as a category of death by physicians reviewing the RHIME, we stratified our multivariate analysis by final category of death being injury versus all other causes. When controlling for death due to injury, there was no longer a significant association between death being outside of the home or health care centre and increased physician agreement in any age category.

**Table 5:** Mutlivariate logistic regression by age category.

	Neonates <29 days		Infants 29d to <1 yr		Children 1 to 4 yrs		Children 5 to 14 yrs	
Variables	OR	(95% CI)	OR	(95% CI)	OR	(95% CI)	OR	(95% CI)
Live With								
Yes	1.00		1.00		1.00		1.00	
No	0.98	(0.86–1.11)	0.99	(0.82–1.18)	1.11	(0.93–1.33)	1.25	(1.00–1.55)
Religion								
Hindu	1.00		1.00		1.00		1.00	
Muslim	0.97	(0.85–1.09)	1.04	(0.87–1.25)	1.00	(0.86–1.17)	0.84	(0.67–1.04)
Other	0.95	(0.78–1.16)	0.87	(0.65–1.18)	0.68	(0.51–0.89)	1.02	(0.73–1.41)
Respondent Education								
<Primary	1.00		1.00		1.00		1.00	
Primary	1.01	(0.88–1.15)	1.10	(0.91–1.34)	0.93	(0.77–1.12)	0.91	(0.71–1.17)
>Primary	1.04	(0.94–1.15)	1.16	(1.00–1.36)	1.08	(0.93–1.26)	0.99	(0.81–1.21)
Respondent Relationship								
Parent	1.00		1.00		1.00		1.00	
Sibling	0.99	(0.73–1.35)	1.07	(0.70–1.62)	1.10	(0.82–1.47)	0.94	(0.71–1.25)
Grandparent	1.14	(1.02–1.27)	1.02	(0.66–1.57)	1.30	(0.94–1.78)	0.96	(0.66–1.39)
Other Relative	1.11	(1.00–1.24)	1.03	(0.67–1.59)	1.11	(0.81–1.50)	0.85	(0.61–1.18)
Neighbour	1.19	(0.95–1.49)	1.08	(0.64–1.84)	1.23	(0.85–1.79)	0.79	(0.51–1.23)
Respondent Gender								
Male	1.00		1.00		1.00		1.00	
Female	1.09	(1.00–1.18)	1.02	(0.89–1.16)	1.06	(0.95–1.19)	0.91	(0.78–1.06)
Death Place								
Home	1.00		1.00		1.00		1.00	
Health Centre	0.92	(0.82–1.03)	1.10	(0.89–1.36)	1.13	(0.93–1.37)	1.07	(0.87–1.32)
Other	0.94	(0.77–1.15)	0.81	(0.64–1.04)	1.66	(1.31–2.10)	2.71	(2.07–3.55)
Deceased Sex								
Male	1.00		1.00		1.00		1.00	
Female	1.05	(0.96–1.14)	0.97	(0.85–1.10)	1.06	(0.95–1.19)	0.80	(0.69–0.93)
Death Registered								
No	1.00		1.00		1.00		1.00	
Yes	0.91	(0.80–1.03)	0.82	(0.67–1.01)	0.90	(0.80–1.01)	0.89	(0.70–1.13)

Footnote: Results are adjusted for region, EAG, urban/rural, and language.

A total of 1726 neonatal deaths and 3922 childhood deaths with causes of death reported by household members (Form 12) could be matched to specific deaths recorded by RHIME (Table S2). Overall, the sensitivity of the household member's determined cause of death compared to the final RHIME cause of death was very poor. For neonates, the category of vaccine preventable diseases had a sensitivity of 65.6% (95% CI 58.4–72.1), however, the remainder of the categories had sensitivities of less than 32%. Similar to our other analyses, the estimated sensitivities did increase with age, however, in general remained poor. The only categories with sensitivities greater than 50% were diarrheal diseases (53.8% (95% CI 49.2–58.1) and 58.2% (95% CI 51.4–64.7) for ages 1 to 4 years and 5 to 14 years) and injuries (60.9% (95% CI 52.9–68.4) and 67.1% (95% CI60.9–72.7) for ages 1 to 4 years and 5 to 14 years).

## Discussion

The purpose of this analysis is to better understand the factors that contribute to physician agreement or disagreement in the determination of cause of death using verbal autopsy. In the Million Death Study, internal processes such as attempted physician reconciliation through the exchange of identified key clinical words, and, if required, adjudication by a third, senior physician, result in a single final identified cause of death. However, identifying specific situations in which trained physicians have more difficulty determining a common cause of death will allow for the tailoring of the VA method to function better in these conditions. We used multiple methods including the kappa statistic, a sensitivity and specificity analysis, and both univariate and multivariate logistic regression to analyze the physician coded causes of death. Though many VA studies use multiple physicians to review VA forms and various reconciliation and adjudication steps to deal with differences in interpretation, to the best of our knowledge, this study is the first to specifically investigate factors that may contribute to physician disagreement.

We were particularly interested in whether specific factors regarding the characteristics of the death itself or of the RHIME respondent would be associated with higher rates of physician disagreement in cause of death assignment. Reassuringly, with few exceptions, the kappa analysis did not uncover specific factors associated with increased physician agreement and the values generally fell within the same category of strength of agreement. Of particular importance is that when taking into account confounding factors in the multivariate analysis, we did not find consistent significant differences in physician agreement based on the death being in a rural or urban area, at home or in a health care facility, registered or not, or an the respondent's gender, religion, relationship to the deceased, or whether or not the respondent lived with the deceased. The similar levels of agreement across these variables is reassuring to all VA based research.

### Age

The likelihood of physician agreement increased with age. The lower level of physician agreement for neonates is not unexpected and is reflective of the fact that many neonatal conditions are relatively non-specific and with a great deal of overlap in clinical presentation. Previous studies [23]outline the gaps in current VA methods in determining neonatal causes of death and our results also suggest physicians using VA data have greater difficulty in coming to a common cause of death in neonates than in older children. Similarly, the ability of physicians to concur on cause of death improves with increasing age in the non-neonatal group as well as seen with the increasing kappa statistic in the childhood age categories. In contrast to neonates, there is likely less overlap in clinical presentation of causes and this suggests that the increased levels of physician agreement may be due to higher quality and more specific information gathered by the RHIME in older children.

### Gender

We also found that the kappa statistic was significantly lower when the deceased was female in both the 1 to 4 years and 5 to 14 years categories. The agreement was also lower in female deaths aged 29 days to 1 year, however, this difference did not reach significance. The univariate and multivariate logistic regressions, the latter of which adjusts for the effect of the other factors, also found lower agreement for female deaths in the 5 to 14 age category. The lower agreement on cause of death assignment in female children may be reflective of a lower quality narrative given for female deaths or captured by the interviewer. Evidence suggests that there is a significant gender bias against female children in India that can result in neglect of girls, death, or selective abortion [24], [25]. Such a bias may also be resulting in less substantive and accurate information accrual by the RHIME and, hence, more difficulty in physicians reviewing the RHIME information determining a cause of death among girls.

### Regional/Poverty Associated Variation

In 2001, eight Indian states that have been lagging behind in containing population growth within manageable limits were classified as part of the Empowered Action Group (EAG). These states are Bihar, Chattisgarh, Jharkhand, Madhya Pradesh, Orissa, Rajasthan, Uttar Pradesh, and Uttaranchal. For this analysis, we have also included the poor state of Assam with this group. It has been reported that neonatal mortality rates are higher in the EAG states than elsewhere in India [26]. We did not see consistent associations between level of agreement and EAG state, geographic region, language of the RHIME, or urban versus rural location. However, we did include these variables in the multivariable model in order to adjust for their effects.

### Household Determination of Death versus Verbal Autopsy

The poor sensitivity and specificity for the laypeople and trained physicians versus the final RHIME category of death as the gold standard reiterates the usefulness and importance of physician coding in determining cause of death in VA studies. Unsurprisingly, with no formal training, the layperson respondents were not able to accurately identify a cause of death compared to trained physicians using the RHIME.

### Limitations

While we examined in great detail the various factors regarding the RHIME respondents and the deceased, we did not analyze the impact of the trained physicians themselves. We have assumed all physicians are equal in terms of training, experience, knowledge, skill, and other factors and thus that each has a similar ability to take information from the RHIME and arriving at a cause of death. In reality, it is certain that this assumption is not true. Future work by our group may further examine the impact of individual physician specialty, years experience, training, and other factors on agreement/disagreement.

The calculated sensitivities and specificities for physician identified causes of death must be interpreted with the understanding that the final RHIME cause of death is not independent from the cause of death assigned by the physicians at the earlier stages. However, this particular analysis is useful in highlighting which groups of causes of death produce more difficulties in achieving physician consensus. Specifically, in neonates the categories of asphyxia and birth trauma, and in non-neonates the categories of central nervous system infections, nutritional diseases, and other infectious diseases had less than a 80% sensitivity in comparison to the final RHIME category of death. This is again likely reflective of inherent difficulties in the RHIME differentiating these relatively clinical non-specific entities from other causes of death.

The level of physician agreement is dependent upon the number of categories being compared. By increasing the number of categories, the level of agreement will decrease and vice versa. The ICD-10 has over 2000 diagnostic codes, many of which require advanced laboratory, microbiologic, or radiologic tools to diagnose. Clearly, the RHIME is not able to classify causes of death to such a level of specificity. In the Million Death Study, the RHIME is designed to code to the first three digits of the ICD-10 codes. In this analysis, we have further collapsed the codes into a smaller number of categories. We chose these categories based on two major factors: one, they make up the majority of mortality in the respective age groups, and two, they are specific enough to be of great utility for informed public health decision making. We feel that whereas it would be ideal for physician reviewers of RHIME to agree on very specific causes of death (for example pneumococcal pneumonia or herpes simplex encephalitis), in reality, and in the absence of sophisticated diagnostic tools, it is sufficient to agree on the broad categories of acute respiratory infection or central nervous system infection. A drawback to our classification system, however, is that no inferences can be made on the ability of physicians use RHIME to come to a common cause of death for the specific and varied causes in the heterogeneous ‘other perinatal conditions’ and ‘other non-infectious conditions’ categories.

### Conclusion

The VA is an invaluable tool in understanding causes of death in settings lacking comprehensive and accurate vital event monitoring systems. Factors influencing physician agreement/disagreement to the greatest degree are the gender and age of the deceased; specifically, physicians tend to be less likely to agree on a common category of death in female children and in younger ages, particularly neonates. Additional training of physician reviewers and continued adaptation of the VA itself, with a focus on gender and age of the deceased, may be useful in increasing rates of physician agreement in these groups. Our study contributes to a better understanding of the factors influencing the VA ability to accurately determine cause of death, and to this end, may serve to promote informed health policy decisions in these settings.

## Supporting Information

Table S1 Strength of agreement of the kappa coefficient (19). Footnote: We recognize that this guideline is somewhat arbitrary and that the magnitude of the kappa value is dependent on both the number of categories and the number of observations. We present the guidelines for comparison purposes only.
(0.03 MB DOC)

Table S2 Sensitivity and specificity for deaths; Form 12* versus RHIME#. Footnotes: * Deaths reported by household members. # Routine, Reliable, Representative and Re-sampled Household Investigation of Mortality with Medical Evaluation.
(0.10 MB DOC)
