# Supplementary material for: Factors Associated with Physician Agreement on Verbal Autopsy of over 27000 Childhood Deaths in India
Source: PLoS One. 2010 Mar 8;5(3):e9583. doi: 10.1371/journal.pone.0009583 (PMC2833201; doi:10.1371/journal.pone.0009583)
Supplement: Table S1 — Strength of agreement of the kappa coefficient (19). Footnote: We recognize that this guideline is somewhat arbitrary and that the magnitude of the kappa value is dependent on both the number of categories and the number of observations. We present the guidelines for comparison purposes only. (0.03 MB DOC) [file pone.0009583.s001.doc]

| Kappa | Strength of Agreement |
| --- | --- |
| <0 | No Agreement |
| 0.0-0.20 | Slight Agreement |
| 0.21-0.40 | Fair Agreement |
| 0.41-0.60 | Moderate Agreement |
| 0.61-0.80 | Substantial Agreement |
| 0.81-1.00 | Almost Perfect Agreement |
