# Supplementary material for: Factors Associated with Physician Agreement on Verbal Autopsy of over 27000 Childhood Deaths in India
Source: PLoS One. 2010 Mar 8;5(3):e9583. doi: 10.1371/journal.pone.0009583 (PMC2833201; doi:10.1371/journal.pone.0009583)
Supplement: Table S2 — Sensitivity and specificity for deaths; Form 12* versus RHIME#. Footnotes: * Deaths reported by household members. # Routine, Reliable, Representative and Re-sampled Household Investigation of Mortality with Medical Evaluation. (0.10 MB DOC) [file pone.0009583.s002.doc]

| **Age Category** | **Category of Death** | **VA Deaths** | **Form 12 Deaths** |  | **Sens.** | **95% CI** |  | **Spec.** | **95% CI** |
| --- | --- | --- | --- | --- | --- | --- | --- | --- | --- |
| **0-28 days** | **Low birth weight/Pre-term** | **443** | **132** |  | **14.7** | **(11.7-18.3)** |  | **94.8** | **(93.4-95.9)** |
|  | **Other Infections** | **488** | **298** |  | **31.1** | **(27.2-35.4)** |  | **88.2** | **(86.3-89.9)** |
|  | **Birth Asphyxia and Birth Trauma** | **315** | **269** |  | **26.3** | **(21.8-31.5)** |  | **86.8** | **(85.0-88.5)** |
|  | **Other Perinatal Conditions** | **185** | **352** |  | **28.6** | **(22.6-35.5)** |  | **80.6** | **(78.6-82.5)** |
|  | **Vaccine Preventable Diseases** | **180** | **289** |  | **65.6** | **(58.4-72.1)** |  | **88.9** | **(87.3-90.4)** |
|  | **Congenital Anomalies** | **57** | **126** |  | **19.3** | **(11.1-31.3)** |  | **93.1** | **(91.8-94.2)** |
|  | **Diarrheal diseases** | **58** | **260** |  | **20.7** | **(12.3-32.8)** |  | **85.1** | **(83.3-86.8)** |
|  |  |  |  |  |  |  |  |  |  |
| **29 days to < 1 year** | **Respiratory Infections** | **332** | **282** |  | **44.9** | **(39.6-50.3)** |  | **79.3** | **(76.1-82.3)** |
|  | **Other Non-infectious** | **194** | **169** |  | **29.4** | **(23.4-36.1)** |  | **85.7** | **(83.1-87.9)** |
|  | **Diarrrheal Diseases** | **200** | **231** |  | **42.5** | **(35.9-49.4)** |  | **81.2** | **(78.3-83.8)** |
|  | **Injuries** | **26** | **38** |  | **34.6** | **(19.4-53.8)** |  | **96.9** | **(95.7-97.9)** |
|  | **Other Infectious Diseases** | **60** | **60** |  | **10.0** | **(4.6-20.1)** |  | **94.1** | **(92.4-95.1)** |
|  | **Malaria** | **16** | **31** |  | **31.3** | **(14.1-55.6)** |  | **97.3** | **(96.1-98.1)** |
|  | **Vaccine Preventable Diseases** | **68** | **85** |  | **44.1** | **(32.9-55.9)** |  | **93.9** | **(92.2-95.2)** |
|  | **Nutritional Diseases** | **55** | **80** |  | **36.4** | **(24.9-49.6)** |  | **93.5** | **(91.7-94.9)** |
|  | **CNS Infections** | **25** | **0** |  | **0.0** |  |  | **100.0** |  |
|  |  |  |  |  |  |  |  |  |  |
| **1 to 4 years** | **Respiratory Infections** | **464** | **447** |  | **46.8** | **(42.3-51.3)** |  | **83.8** | **(81.8-85.7)** |
|  | **Other Non-infectious** | **275** | **191** |  | **29.8** | **(24.7-35.5)** |  | **93.2** | **(91.9-94.4)** |
|  | **Diarrrheal Diseases** | **442** | **561** |  | **53.8** | **(49.2-58.4)** |  | **77.6** | **(75.4-79.7)** |
|  | **Injuries** | **151** | **138** |  | **60.9** | **(52.9-68.4)** |  | **97.3** | **(96.6-98.1)** |
|  | **Other Infectious Diseases** | **129** | **151** |  | **17.8** | **(12.2-25.3)** |  | **92.7** | **(91.4-93.8)** |
|  | **Malaria** | **79** | **76** |  | **25.3** | **(17.0-35.9)** |  | **96.9** | **(96.0-97.6)** |
|  | **Vaccine Preventable Diseases** | **182** | **202** |  | **40.7** | **(33.8-47.9)** |  | **92.5** | **(91.1-93.7)** |
|  | **Nutritional Diseases** | **94** | **120** |  | **34.0** | **(25.3-44.1)** |  | **95.1** | **(94.0-96.0)** |
|  | **CNS Infections** | **70** | **0** |  | **0.0** |  |  | **100.0** |  |
|  |  |  |  |  |  |  |  |  |  |
| **4 to 14 years** | **Respiratory Infections** | **106** | **129** |  | **40.6** | **(31.7-50.1)** |  | **91.0** | **(88.9-92.6)** |
|  | **Other Non-infectious** | **239** | **192** |  | **42.7** | **(36.6-49.0)** |  | **89.0** | **(86.7-91.0)** |
|  | **Diarrrheal Diseases** | **208** | **292** |  | **58.2** | **(51.4-64.7)** |  | **79.9** | **(77.1-82.5)** |
|  | **Injuries** | **240** | **197** |  | **67.1** | **(60.9-72.7)** |  | **95.6** | **(93.9-96.8)** |
|  | **Other Infectious Diseases** | **72** | **116** |  | **25.0** | **(16.4-36.1)** |  | **90.1** | **(88.1-91.8)** |
|  | **Malaria** | **48** | **45** |  | **20.8** | **(11.4-34.2)** |  | **96.5** | **(95.2-97.5)** |
|  | **Vaccine Preventable Diseases** | **63** | **62** |  | **41.3** | **(29.9-53.6)** |  | **96.4** | **(95.1-97.4)** |
|  | **Nutritional Diseases** | **11** | **26** |  | **18.2** | **(5.1-47.7)** |  | **97.7** | **(96.6-98.5)** |
|  | **CNS Infections** | **72** | **0** |  | **0.0** |  |  | **100.0** |  |
